# Viral kinetics, stability and sensitivity analysis of the within-host COVID-19 model

**DOI:** 10.1038/s41598-023-38705-6

**Published:** 2023-07-19

**Authors:** Islam M. Elbaz, H. El-Metwally, M. A. Sohaly

**Affiliations:** 1 Faculty of Basic Sciences, Galala University, Suez, 435611 Egypt; 2 Faculty of Energy and Environmental Engineering, British University in Egypt, Cairo, 11837 Egypt; 3 Mathematics Department, Faculty of Science, Mansoura University, Mansoura, 35516 Egypt

**Keywords:** Applied mathematics, Listeria, Infectious diseases

## Abstract

This paper delves into the investigation of the COVID-19 dynamics within a host using the Target-Latent-Infected-Virus (TLIV) model, presenting a fresh approach compared to previous studies. Our model introduces a latent class and explores sensitivity analysis, aspects that have received limited attention in prior research. A significant contribution of this study is the analysis of both local and global stability of equilibrium states, subject to specific sufficient conditions based on the basic reproduction number $R_0$. By examining these stability properties, we aim to gain insights into the factors underlying variations observed in the findings of different studies. Additionally, we identify the death rate of infected cells as the parameter most susceptible to influence in our model. To minimize its impact and facilitate recovery, it is crucial to implement appropriate medical therapies and consume immune-boosting foods. Some computer simulations are carried out to strengthen the theoretical results.

## Introduction

The novel coronavirus SARS-CoV-2, emerged as a global health crisis in history that has been uncovered by the year 2020. In Wuhan, Hubei Province, China in December 2019, coronavirus (COVID-19) disease was first reported^1^. The virus affected more than two hundred countries and killed millions of people according to the World Health Organization. The infection can be controlled by physical social distancing, self-isolation at home, face masks, hand-washing, and surface cleaning^2,3^. Several countries have proposed strict social distancing and lock-down regulations to stop the spread of the virus. It is primarily transmitted through respiratory droplets when an infected individual coughs, sneezes, talks, or breathes. The virus can also spread by touching contaminated surfaces and then touching the face. Common symptoms of COVID-19 include fever, cough, fatigue, shortness of breath, and loss of taste or smell, although asymptomatic cases are also prevalent^4^.

The disease severity ranges from mild to severe, with some cases progressing to acute respiratory distress syndrome and multi-organ failure, particularly in vulnerable populations such as the elderly and those with underlying health conditions^5,6^. Rapid and widespread transmission led to the declaration of a pandemic by the World Health Organization (WHO) in March 2020, prompting the implementation of various control measures like social distancing, mask-wearing, and vaccination campaigns^7^. Efforts to combat the disease have also focused on testing, contact tracing, and the development and distribution of vaccines. Understanding the basic details of COVID-19 is crucial for effectively managing and mitigating its impact on public health systems and communities worldwide.

Mathematical epidemiological models play a crucial role in understanding the dynamics of infectious diseases, providing valuable insights into how these diseases spread within populations. These models capture the rapid fluctuations in the number of infected individuals, serving as the fundamental principle of mathematical modeling. A wide range of mathematical models that describe various types of diseases can be found in the literature^8–14^. Numerous studies with many intriguing mathematical models that have examined the dynamic behavior of SARS-CoV-2 can be found in^2,15,16^.

Within the human body, biological mathematical models offer valuable insights into the complex dynamics of infectious diseases at an individual level. These models integrate principles from both biology and mathematics to depict the interplay between pathogens, the immune system, and host cells^17^. By considering factors such as viral replication, dynamics of immune responses, and the effects of treatments, these models facilitate a deeper comprehension of disease progression and contribute to informed therapeutic interventions^18^.

Few research papers could predict the dynamics of COVID-19 disease accurately and according to World Health Organization, dozens of vaccine candidates are in clinical research, and about ten vaccines are authorized for public use^19,20^. Clinically, there is no effective treatment that can remove the virus from the human body, however, the available treatments help like Ebola, Influenza, and SARS-CoV-1.

It is known that several works have focused on forecasting the number of infected individuals in populations^21,22^. Forecasting for COVID-19 is exceedingly difficult and has failed in many papers because of the type of mathematical models, missing data, and/or the random behavior of this virus^23^. We think it is time to study the dynamics of COVID-19 within-host instead of between the human populations.

### Within-host COVID-19 model

Based on current data, it is estimated that around 80% of COVID-19 cases are classified as mild to moderate, and individuals in these cases typically experience a full recovery from the infection^24^. Previous research has indicated that the humoral response, involving the production of antibodies, to SARS-CoV-2 infection is commonly observed in individuals who have been infected. The level of antibodies, specifically the anti-SARS-CoV-2 IgG titers, has been strongly linked to the extent of virus-specific CD4+ and CD8+ T cell responses in the bloodstream^25^. However, it should be noted that most samples of convalescent plasma, collected from individuals recovering from COVID-19, did not exhibit high levels of neutralizing activity. Furthermore, only a small number of individuals who were analyzed showed the presence of rare antibodies that possess potent antiviral activity against specific viral proteins^26^.

The angiotensin-converting enzyme 2 (ACE2) receptors present on the surface epithelial cells are bound by the SARS-CoV-2 virus. These cells, which express ACE2, are considered susceptible to viral infection and are referred to as target cells in mathematical models. The distribution of these target cells varies notably across different sections of the respiratory system, with the lungs having the highest density, followed by the nose, and finally, the tissues of the trachea/bronchi. Consequently, COVID-19 patients often experience pneumonia, making it a relatively frequent occurrence^27,28^.

Our proposed model in this paper comprises four variable quantities, the susceptible targeted cells, *T*(*t*), latent cells, *L*(*t*), infected cells, *I*(*t*), and free virus particles, *V*(*t*). The model assumes that there is a constant of regeneration $d_1 T(0)$ susceptible targeted cells. These cells are infected by free virus particles with a bilinear incidence rate $\beta T(t)V(t)$ and these infected cells produce with a rate *p* free virus particles. Parameters $d_1, \, d_2, \, d_3,$ and $d_4$ are the death rate of susceptible target cells, latent cells, infected cells, and free virus particles, respectively. Latent cells on average span 1/*k* units of time in *L* class and then join the infected class of cells. It should be noted that $d_1$ is a natural death rate or natural clearance rate while $d_2, \, d_3$ and $d_4$ are a combination of the natural clearance rate and the role of the immune system in the elimination of these cells.

Our work presents a novel mathematical model that incorporates latent class analysis to describe the dynamics of COVID-19 within the host. Unlike previous studies that focused solely on estimating the basic reproductive number^29^, or analyzing the stability of equilibrium states^30^, our model delves deeper into the impact of various parameters on the system. By conducting sensitivity analysis, we have identified the death rate of infected cells as the most sensitive parameter in our model. This finding sheds light on the critical role this parameter plays in shaping the dynamics of COVID-19 infection and highlights its potential implications for disease progression and management strategies.

Authors in^29,30^ have studied the viral kinetics of COVID-19 without latent class of cells, we consider the mathematical within-host model in the form

1 $$\begin{aligned} \begin{aligned} {\dot{T}}(t)&= d_1 T(0) - \beta T(t) V(t) - d_1 T(t), \\ {\dot{L}}(t)&= \beta T(t) V(t) - (d_2 + k) L(t), \\ {\dot{I}}(t)&= k L(t) - d_3 I(t), \\ {\dot{V}}(t)&= p I(t) - d_4 V(t). \end{aligned} \end{aligned}$$

The originality of our model lies in the inclusion of a new class, namely the latent cells, to capture the dynamics of COVID-19 within the human body. Within this class, individuals or cells have been exposed to the disease but have not yet reached the infectious state. The duration of this latent period, determined by the delay parameter, signifies the time it takes for individuals or cells to become infectious. This approach is applicable to more general fractional systems, see^31^.

In the traditional model, only the susceptible, infected, and viral load compartments were considered. However, by introducing the latent class, we aim to provide a more realistic representation of the disease progression. This addition accounts for the incubation period during which the virus replicates within the body before symptoms become apparent. By incorporating the latent class, our model captures an essential aspect of the disease phenomenon, enhancing its accuracy and predictive capabilities. It can serve as a valuable tool for studying the effectiveness of interventions and informing public health strategies aimed at controlling the spread of COVID-19.

Here are the model assumptions:

1. In COVID-19, the respiratory system’s cell population remains constant due to the slow turnover of respiratory epithelial cells.
2. $d_1T(0)$ is assumed to be a constant of regeneration of cells.
3. Once cells recover from the infection and become immune, the model assumes that they remain immune indefinitely.
4. Cell variations in susceptibility are not considered.
5. During the latent period, cells are considered non-infectious or have a low probability of transmitting the disease to other cells.
6. There are no major changes in the characteristics or behavior of cells within the latent class during the modeling period.

### Graph of the system

**Figure 1:**
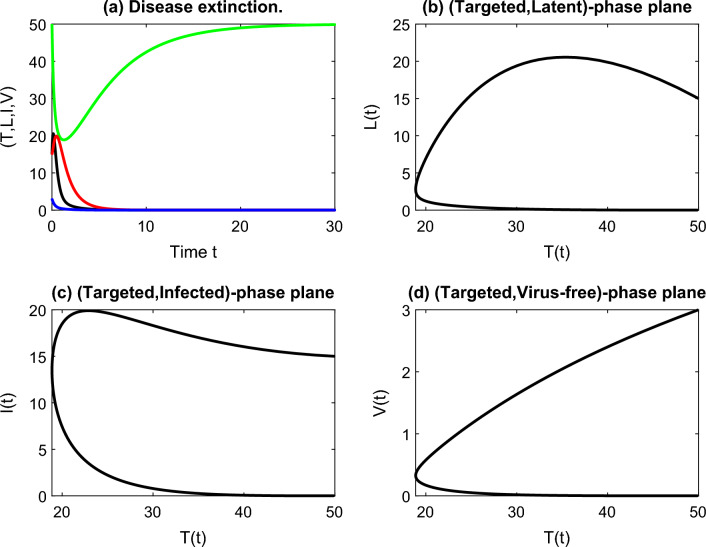
Graph of the cell populations shows the extinction of infected cells and virus-free particles over time with phase planes that show the behavior of each compartment with the targeted cells.

By performing parameter estimation in a similar way to^29,30^, using the Monte Carlo Markov Chain (MCMC) method, and in light of the chest radiograph score data, the estimated values of parameters are $$T(0) = 50, \, \beta = 1, \, d_1 = 0.2, \, d_2 = 2, d_3 = 1.1, \, d_4 = 3, \, k = 1.7$$ and $$ p = 0.05$$. In this scenario, the graph of our model (1) can be sketched in Fig. 1 in 30 days.

Basic properties of the system like the nonnegativity and uniform boundedness of the solution are shown in the next section. The equilibrium states of the model and the basic reproduction number are provided in Section “Equilibrium states and the basic reproduction number”. Local and global stability of the equilibrium states $E_0$ and $E^*$ with some computer simulations are shown in Section “Extinction and persistence”. Viral kinetics of the COVID-19 model using sensitivity and elasticity analysis of $R_0$ towards the effective parameters in the model are shown in Section “[Sec Sec9]”. Conclusions and some future works are devoted to Section “Conclusion and further directions.

## Nonnegativity and uniform boundedness of the solutions of (1)

In this section, we study the nonnegativity and the uniform boundedness of the solutions of the within-host model (1) with respect to the initial conditions

2 $$\begin{aligned} \left( T(0), L(0), I(0), V(0) \right) \in {\mathbb {R}}_+^4. \end{aligned}$$

The next theorem proving nonnegativity and uniform boundedness in the mathematical model of cell population dynamics ensures valid interpretations and prevents unrealistic predictions, supporting reliable analysis and decision-making in cell biology and related fields.

### Theorem 2.1

*Assume that the initial values* (2), *then all solutions of* (1) *are nonnegative and uniformly bounded*.

### Proof

The first equation of model (1) is

$$\begin{aligned} {\dot{T}}(t) = d_1 T(0) - \left( \beta V(t) + d_1 \right) T(t), \end{aligned}$$

Using the integrating factor

$$\begin{aligned} e^{\int _{0}^{t} \left( \beta V(s) + d_1 \right) ds}, \end{aligned}$$

we have

$$\begin{aligned}{} & {} {\dot{T}}(t) e^{\int _{0}^{t} \beta V(s) ds + d_1 t} \ge d_1 T(0) e^{\int _{0}^{t} \beta V(s) ds + d_1 t}, \\{} & {} T(t) e^{\int _{0}^{t} \beta V(s) ds + d_1 t} \ge T(0) \int _{0}^{t} d_1 T(0) e^{\int _{0}^{\tau } \beta V(s) ds + d_1 t} d\tau , \end{aligned}$$

i.e.,

$$\begin{aligned}{} & {} T(t) \ge T(0) e^{-\int _{0}^{t} \beta V(s) ds + d_1 t} + e^{-\int _{0}^{t} \beta V(s) ds + d_1 t} \int _{0}^{\tau } d_1 T(0) e^{\int _{0}^{\tau } \beta V(s) ds + d_1 t} d \tau > 0. \end{aligned}$$

Similarly,

$$\begin{aligned} \begin{aligned} L(t)&= L(0) e^{-\int _{0}^{t} (d_2 + k) ds} + e^{-\int _{0}^{t} (d_2 + k) ds} \int _{0}^{t} \beta T(\tau ) V(\tau ) e^{\int _{0}^{\tau } (d_2 + k) ds} d \tau> 0, \\ I(t)&= I(0) e^{-\int _{0}^{t} d_3 ds} + e^{-\int _{0}^{t} d_3 ds} \int _{0}^{t} k L(\tau ) e^{\int _{0}^{\tau } d_3 ds} d \tau> 0, \\ V(t)&= V(0) e^{-\int _{0}^{t} d_4 ds} + e^{-\int _{0}^{t} d_4 ds} \int _{0}^{t} p I(\tau ) e^{\int _{0}^{\tau } d_4 ds} d \tau > 0. \end{aligned} \end{aligned}$$

Regarding the boundedness of the solutions of (1), the total population of cells $N(t) = T(t)) + L(t) + I(t)$, where

$$\begin{aligned} \begin{aligned} {\dot{N}}(t)&= d_1 T(0) - d_1 T(t) - d_2 L(t) - d_3 I(t) \\&\le d_1 T(0) - \min \lbrace d_1, d_2, d_3\rbrace \left( T(t) + L(t) + I(t) \right) . \end{aligned} \end{aligned}$$

Assume that $d = \min \lbrace d_1, d_2, d_3\rbrace$, then

$$\begin{aligned} {\dot{N}}(t) \le d_1 T(0) - d N(t), \end{aligned}$$

and

$$\begin{aligned} \limsup _{t \rightarrow \infty } N(t) = \frac{d_1 T(0)}{d}. \end{aligned}$$

Consequently, all solutions of (1) with respect to (2) are bounded in a biologically feasible region

$$\begin{aligned} \Gamma = \left\{ \left( T(t), L(t), I(t) \right) \in {\mathbb {R}}_+^3 \Big | T(t) + L(t) + I(t) \le \frac{d_1 T(0)}{d} \right\} . \end{aligned}$$

Clearly, the number of free virus particles is also bounded at any time *t*. $\square$

## Equilibrium states and the basic reproduction number

Equilibrium states of this model satisfy the following algebraic equations

$$ \begin{aligned}d_1 T(0) = \beta T V - d_1 T, \qquad \qquad &\beta T V = (d_2 + k) L(t),\\k L(t) = d_3 I(t), \qquad \qquad &p I(t) = d_4 V(t). \end{aligned}$$

Clearly, we have two equilibrium states at most, the infection-free equilibrium state $E_0 = (T_0,L_0,I_0,V_0) = (T(0),0,0,0)$, and a positive endemic equilibrium state

$$\begin{aligned} \begin{aligned} E^*&= (T^*,L^*,I^*,V^*)\\&= \left( \frac{(d_2 + k)d_3 d_4}{\beta k p}, \frac{d_1 T(0)}{d_2 + k} - \frac{d_1 d_3 d_4}{\beta k p}, \frac{d_1 k T(0)}{d_3 (d_2 + k)} - \frac{d_3 d_4}{\beta p}, \frac{d_1 T(0) k p}{d_3 d_4 (d_2 + k)} - \frac{d_1}{\beta } \right) . \end{aligned} \end{aligned}$$

Using the method of Next Generation Matrix (NGM) used by^32^, we calculate the basic reproduction number $R_0$. The three compartments of infection are

$$\begin{aligned} \dfrac{d}{dt} \left[ \begin{array}{c} L(t) \\ I(t) \\ V(t) \end{array} \right] = \left[ \begin{array}{c} \beta T(t) V(t) - (d_2 + k) L(t) \\ k L(t) - d_3 I(t) \\ p I(t) - d_4 V(t) \end{array} \right] = {\textsf{F}}-{\textsf{V}}, \end{aligned}$$

where

$$\begin{aligned} {\textsf{F}} = \left[ \begin{array}{c} \beta T(t) V(t) \\ 0 \\ 0 \end{array} \right] , \, \, \, \, {\textsf{V}} = \left[ \begin{array}{c} (d_2 + k) L(t) \\ -k L(t) + d_3 I(t) \\ -p I(t) + d_4 V(t) \end{array} \right] . \end{aligned}$$

The Jacobians of ${\textsf{F}}$ and ${\textsf{V}}$ are

$$\begin{aligned} {\textsf{F}}_0 = \left[ \begin{array}{ccc} 0 &{} 0 &{} \beta T_0 \\ 0 &{} 0 &{} 0 \\ 0 &{} 0 &{} 0 \end{array} \right] = \left[ \begin{array}{ccc} 0 &{} 0 &{} \beta T(0) \\ 0 &{} 0 &{} 0 \\ 0 &{} 0 &{} 0 \end{array} \right] , \\ {\textsf{V}}_0 = \left[ \begin{array}{ccc} d_2 + k &{} 0 &{} \beta T_0 \\ -k &{} d_3 &{} 0 \\ 0 &{} -p &{} d_4 \end{array} \right] . \end{aligned}$$

Then

$$\begin{aligned} {\textsf{F}}_0 {\textsf{V}}_0^{-1} = \left[ \begin{array}{ccc} \frac{\beta T(0) k p}{(d_2 + k) d_3 d_4} &{} \frac{\beta T(0)p}{d_3 d_4} &{} \frac{\beta T(0)}{d_4} \\ 0 &{} 0 &{} 0 \\ 0 &{} 0 &{} 0 \end{array} \right] . \end{aligned}$$

The basic reproduction number $R_0$ is defined as the spectral radius of ${\textsf{F}}_0 {\textsf{V}}_0^{-1}$, consequently

$$\begin{aligned} R_0 = \frac{\beta T(0) k p}{(d_2 + k) d_3 d_4}. \end{aligned}$$

Then for $R_0 >1$, there is a unique positive equilibrium that can be written again in the form

$$\begin{aligned} E^*= \left( \frac{1}{R_0}, \frac{d_1 d_3 d_4}{\beta k p} (R_0 - 1), \frac{d_1 d_4}{\beta p}(R_0 - 1), \frac{d_1}{\beta }(R_0 - 1) \right) . \end{aligned}$$

## Extinction and persistence

In this section, we shall perform the stability analysis, in terms of the basic reproduction number $R_0$, we study the extinction and the persistence of the disease inside the human body by focusing on the local and global stability of $E_0$ and $E^*$. Evaluating the Jacobian matrix *J* at the equilibrium states, the LaSalle-invariance principle and the Routh-Hurwitz criterion^33^ are used for investigating the local stability of these equilibrium states. Introducing appropriate Lyapunov functions around the equilibrium states is very helpful in investigating the global stability of these states^34^.

### Extinction

#### Theorem 4.1

*If* $R_0 < 1$, *the infection-free equilibrium* $E_0$ *is locally asymptotically stable. And if* $R_0 \le 1$, *then* $E_0$ *is globally asymptotically stable*.

#### Proof

The Jacobian matrix for the right hand side of (1) is

$$\begin{aligned} J(T,L,I,V) = \left[ \begin{array}{cccc} -\beta V - d_1 &{} 0 &{} 0 &{} -\beta T \\ \beta V &{} -(d + k) &{} 0 &{} \beta T \\ 0 &{} k &{} -d_3 &{} 0 \\ 0 &{} 0 &{} p &{} -d_4 \end{array} \right] . \end{aligned}$$

Evaluating *J* at the infection-free equilibrium $E_{0}$

$$\begin{aligned} J\Big |_{E_0} = \left[ \begin{array}{cccc} -d_1 &{} 0 &{} 0 &{} -\beta T_0 \\ 0 &{} -(d_2 + k) &{} 0 &{} \beta T_0 \\ 0 &{} k &{} -d_3 &{} 0 \\ 0 &{} 0 &{} p &{} -d_4 \end{array} \right] . \end{aligned}$$

Let $\lambda _i$ be the eigenvalues of this matrix for $i = 1,2,3,4$ such that

$$\begin{aligned} \begin{aligned} \sum _{i=1}^{4} \lambda _i&= Tr\left( J\Big |_{E_0} \right) = - \left( d_1 + d_2 + k + d_3 + d_4 \right) < 0 \\ \prod _{i=1}^{4} \lambda _i&= det \left( J\Big |_{E_0} \right) = -d_1 \left( \beta T_0 k p - d_3 d_4 (d_2 + k) \right) \\&= -d_1 d_3 d_4 (d_2+k) \left( \frac{\beta T_0 k p}{d_3 d_4(d_2 + k)} - 1 \right) \\&= -d_1 d_3 d_4 (d_2+k) (R_0 - 1). \end{aligned} \end{aligned}$$

If $R_0 < 1, \, \prod _{i=1}^{4} \lambda _i > 0$, then the infection-free equilibrium state is locally asymptotically stable. For global stability of $E_0$, choose the Lyapunov function in the form

$$\begin{aligned} {\mathcal {V}} = \frac{p k}{d_3 (d_2+k)} L + \frac{p}{d_3} I + V \end{aligned}$$

where

$$\begin{aligned}  \frac{d{\mathcal {V}}}{dt}&= \frac{p k}{d_3 (d_2+k)} (\beta T V - d_2 L - k L) + \frac{p k L}{d_3} - d_4 V \\&= d_4 V \left( \frac{p k \beta T}{d_3 d_4 (d_2+k)} - 1 \right) - \frac{d_2 p k L}{d_3 (d_2+k)} - \frac{p k^2 L}{d_3 (d_2+k)} + \frac{p k L}{d_3} \\&\le d_4 V (R_0 - 1) + \frac{p k L}{d_3} \left( 1 - \frac{d_2}{d_2 + k} - \frac{k}{d_2 + k} \right) \\&= d_4 V (R_0 - 1) \end{aligned}$$

Then $\frac{d{\mathcal {V}}}{dt} < 0$ if $R_0 < 1,$ and $\frac{d{\mathcal {V}}}{dt} = 0$ if only $V = 0$ or $R_0 = 1$. According to the LaSalle-invariance principle^34^, the equilibrium state $E_0$ is globally asymptotically stable. $\square$

The theorem’s implications are significant for disease control strategies, as it offers insights into conditions favoring infection eradication and emphasizes the significance of minimizing the basic reproduction number ($R_0$) to prevent outbreaks and ensure the well-being of the cell population.

The Euler method scheme is utilized for simulating the within-host COVID-19 model^35^. This numerical approach partitions time into small intervals and estimates variable changes within each interval. Through the iterative application of this scheme, the model can simulate disease progression over time, considering parameters such as viral replication, immune response, and cell death rates. Other numerical schemes have also been employed to simulate COVID-19 and other various disease models within-host and between individuals^36–38^.

**Figure 2:**
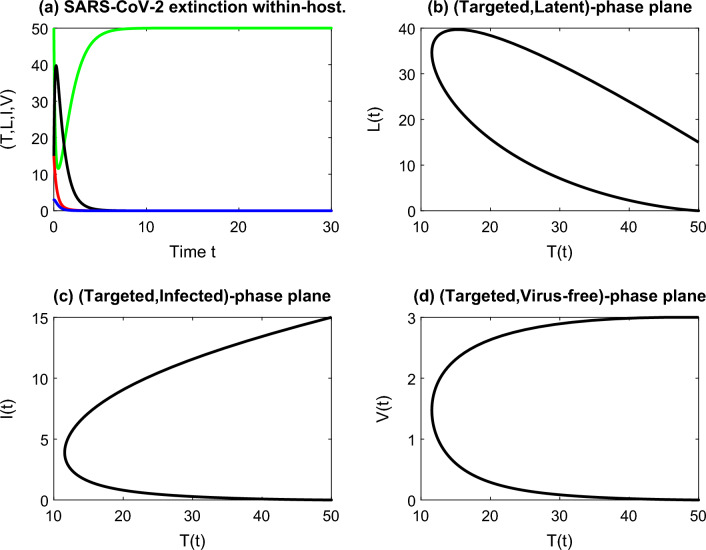
(**a**) Stable disease-free equilibrium implies the extinction of the within-host COVID-19 with $R_0 = 0.2415 < 1$. The behavior of the cell classes with the targeted class is shown in graphs (**b**–**d**).

Using the initial values $(T(0),L(0),I(0),V(0))=(50,15,15,3)$ and the parameters $\beta = 2, \, d_1 = 0.9, \, d_2 = 1.8, \, d_3 = 3.1, \, d_4 = 4.5, k = 0.07$ and $p = 0.9$, we can see the convergence of the trajectory of the solution to the infection-free equilibrium state. Infected cells, Latent cells, and free virus particles will be eliminated from the human body for $R_0 < 1$ as shown in Fig. 2a. The relations between target cells and the other compartments are shown by the phase planes in Fig. 2b–d. The disease dies out within the human body for $R_0 < 1$.

### Persistence

#### Theorem 4.2

*The positive endemic equilibrium* $E^*$ *is locally asymptotically stable and globally asymptotically stable if* $R_0 > 1$.

#### Proof

Evaluating *J* at the positive endemic equilibrium implies

$$\begin{aligned} J\Big |_{E^*} = \left[ \begin{array}{cccc} -\beta V^*- d_1 &{} 0 &{} 0 &{} -\beta T^*\\ \beta V^*&{} -(d_2 + k) &{} 0 &{} \beta T^*\\ 0 &{} k &{} -d_3 &{} 0 \\ 0 &{} 0 &{} p &{} -d_4 \end{array} \right] . \end{aligned}$$

The characteristic equation is

$$\begin{aligned} \begin{aligned} \lambda ^4&+ \underbrace{ \left( \beta V^*+ k + d_1 + d_2 + d_3 + d_4 \right) }_{a_1} \lambda ^3 \\&+ \underbrace{\left( (\beta V^*+ k + d_1 + d_2 + d_4)d_3 + (\beta V^*+ k + d_1 + d_2)d_4 + (d_2 + k)(\beta V^*+ d_1) \right) }_{a_2} \lambda ^2 \\&+ \underbrace{ \left( ((\beta V^*+ d_1 + d_2 + k)d_4 + (d_2 + k)(\beta V^*+ d_1) )d_3 + (d_2 + k)(\beta V^*+ d_1) - k \beta p T^*\right) }_{a_3} \lambda \\&+ \underbrace{ (d_2 + k)(\beta V^*+ d_1)d_3 d_4 - \beta d_1 k p T^*}_{a_4} = 0. \end{aligned} \end{aligned}$$

Now, for $$R_0 > 1,$$

$$\begin{aligned}\Delta _1 &= |a_1| = \beta V^*+ k + d_1 + d_2 + d_3 + d_4 = \beta \left( \frac{d_1}{\beta }(R_0-1) + k + d_1 + d_2 + d_3 + d_4 \right)> 0. \\ \Delta _2&= \begin{vmatrix} a_1&a_3 \\ a_0&a_2 \end{vmatrix} = a_1 a_2 - a_3 > 0, \end{aligned}$$

and

$$\begin{aligned} \Delta _3 = \begin{vmatrix} a_1&a_3&a_5 \\ a_0&a_2&a_4 \\ 0&a_1&a_3 \end{vmatrix} = a_3 \Delta _2 - a_1^2 a_4 > 0. \end{aligned}$$

Then according to the Routh–Hurwitz criterion, the positive endemic equilibrium $E^*$ is locally asymptotically stable. Regarding the global stability of $E^*$, choose the Lyapunov function

$$\begin{aligned} {\mathcal {V}} = T - T^*- T^*\ln \dfrac{T}{T^*} + L - L^*- L^*\ln \dfrac{L}{L^*} + I - I^*- I^*\ln \dfrac{I}{I^*} + V - V^*- V^*\ln \dfrac{V}{V^*}. \end{aligned}$$

Then

$$\begin{aligned} \begin{aligned} \dot{{\mathcal {V}}}&= d_1 T(0) - d_1 T - d_2 L - d_3 I - d_4 V + p I - d_1 T(0) \frac{T^*}{T} + \beta T^*V + d_1 T^*- \beta \frac{L^*T V}{L} + d_2 L^*\\&\quad + k L^*- k \frac{I^*L}{I} - d_3 I^*- p \frac{V^*I}{V} + d_4 V^*\end{aligned} \end{aligned}$$

Using the equations

$$\begin{aligned} d_1T(0)&= d_1 T^*+ \beta T^*V^*, \\ d_4&= \frac{p I^*}{V^*}, \\ k&= \frac{d_3 I^*}{L^*}, \\ \beta&= \frac{d_2 L^*}{T^*V^*} + \frac{k L^*}{T^*V^*}, \end{aligned}$$

implies

$$\begin{aligned} \begin{aligned} \dot{{\mathcal {V}}}&= 2d_1 T^*- d_1 T - d_1 L - d_3 \frac{(T^*)^2}{T} + p I - \frac{p T^*V}{V^*} + p I^*- p \frac{V^*I}{V} + k L^*- k \frac{I^*L}{I} + 2d_2 L^*\\&\quad + 2d_3 I^*- d_2 L - d_3 I - \frac{T^*}{T}(d_2 L^*+ d_3 I^*) + \frac{d_2 L^*V}{V^*} + \frac{d_3 I^*V}{V^*} - \frac{L^*T V}{L}\left( \frac{d_2 L^*}{T^*V^*} + \frac{d_3 I^*}{T^*V^*}\right) \\&= d_1 T^*\left( 2 - \frac{T}{T^*} - \frac{T^*}{T}\right) + p I \left( 1- \frac{I^*V}{V^*I}\right) + \left( p I^*+ \frac{d_3 I^*V}{V^*}\right) \left( 1- \frac{V^*I}{V I^*}\right) \\&\quad + \left( d_2 L^*+ d_3 I^*\right) \left( 2 - \frac{T^*}{T} - \frac{L^*T V}{L T^*V^*}\right) + k L^*\left( 1 - \frac{I^*L}{L^*I} \right) + \frac{d_2 L^*V}{V^*} \left( 1 - \frac{V^*L}{L^*V} \right) \end{aligned} \end{aligned}$$

As the arithmetic mean is greater than or equal to the geometric mean, then $\dot{{\mathcal {V}}} < 0$ and $\dot{{\mathcal {V}}} = 0$ only if $S = S^*, L = L^*, I = I^*$ and $V = V^*$. Then according to the LaSalle-invariance principle, the positive endemic equilibrium $E^*$ is globally asymptotically stable. $\square$

**Figure 3:**
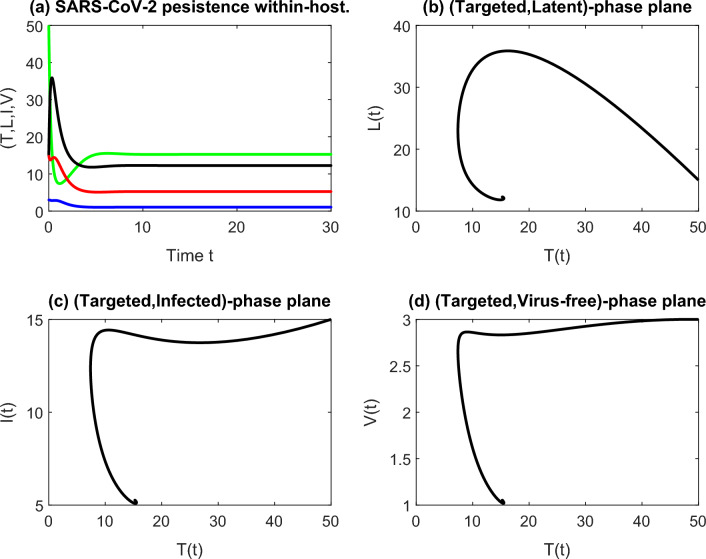
(**a**) Stable endemic equilibrium implies the persistence of the within-host COVID-19 with $R_0 = 3.2773 > 1$. The behavior of the cell classes with the targeted class is shown in graphs (**b**–**d**).

Using the same initial values of the numerical simulation in Fig. 2a, and the parameters $\beta = 1.3, \, d_1 = 0.6, \, d_2 = 0.8, \, d_3 = 2.1, \, d_4 = 4.5, k = p = 0.9$, the disease persists within the human body. Figure3a shows the convergence of the trajectory of the solution towards the positive equilibrium state $E^*$ for $R_0 > 1$ with some phase portraits that show the behavior of the target cells with each compartment in Fig.3b–d.

## Sensitivity and elasticity of $R_0$

**Figure 4:**
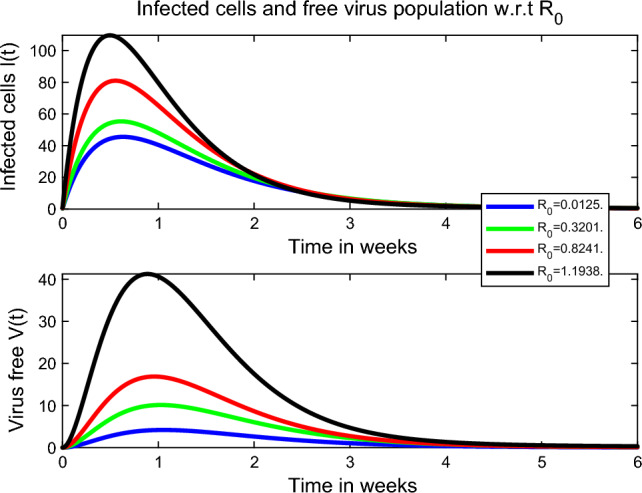
Impact of $R_0$ towards the number of infected cells and free virus particles. Extinction of the disease within-host occurs for smaller values of the basic reproduction number $R_0$.

**Figure 5:**
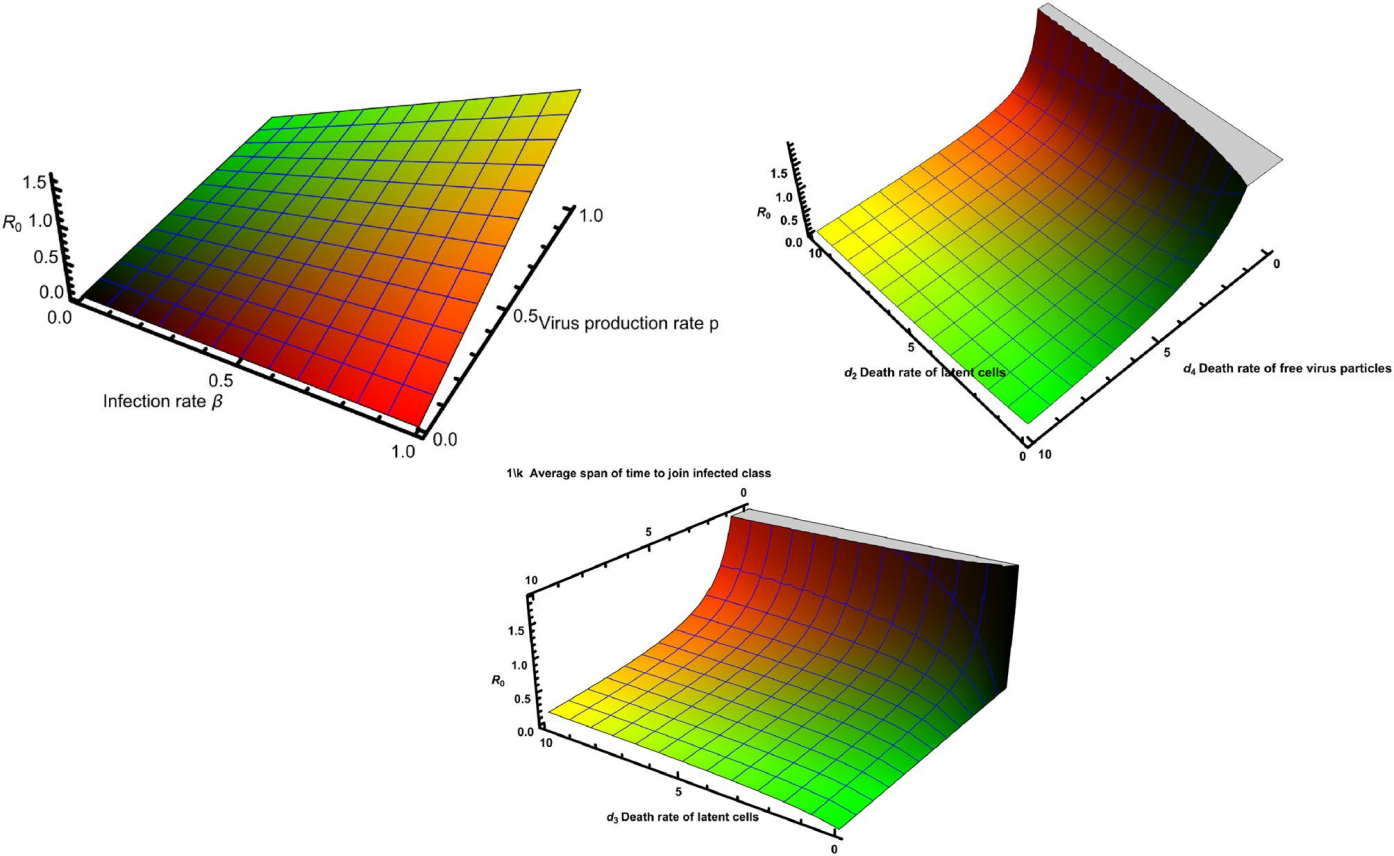
Variation of $R_0$ as a measure of the disease’s potential for spread within a population. The 3D plots show the impact of the parameters o the system on $R_0$.

The basic reproduction number $R_0$ quantifies the average number of secondary cases generated by the introduction of a disease into a susceptible population. It is a vital measure for assessing the potential for disease transmission^39^. Many papers have used optimal control strategies and sensitivity analysis of many disease models including COVID-19, see^40,41^. In our study, we examine the impact of the basic reproduction number $R_0$ on the dynamics of infected cells and free virus particles over a span of six weeks, as illustrated in Fig. 4. During this time interval, we observe that higher values of $R_0$ lead to a greater peak in the number of infected cells and free virus particles. This suggests that a higher $R_0$ is associated with a more pronounced spread of the infection within the host. Furthermore, in Fig. 5, we present surface plots that depict the relationship between $R_0$ and the various parameters incorporated into our model. These plots allow us to visualize how changes in these parameters influence the value of $R_0$, providing insights into the factors that impact the transmissibility and progression of the infection.

**Figure 6:**
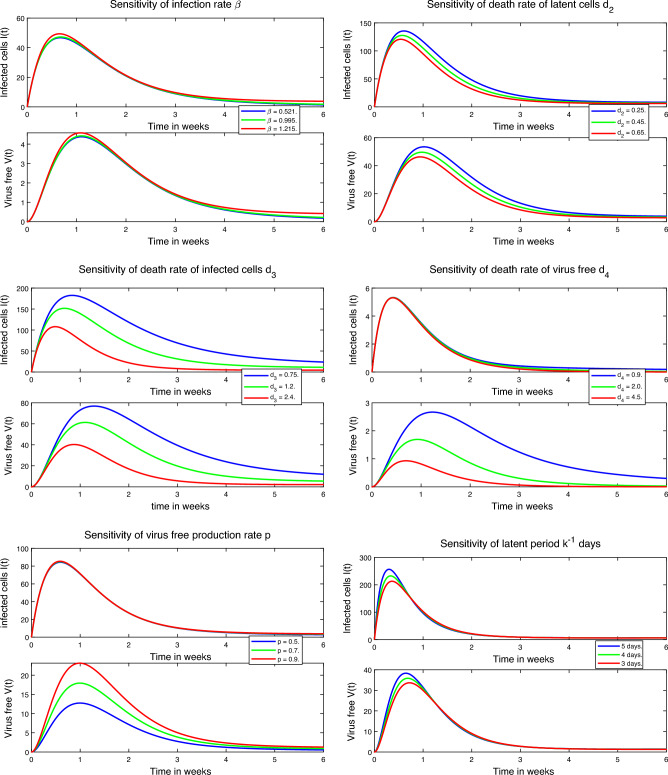
The sensitivity analysis of within-host model parameters involves examining the impact of variations in parameters on the model’s output. By systematically altering these parameters, we can assess their influence on key model outputs such as viral load, disease progression, and the effectiveness of interventions or treatments.

In order to gain insights into the factors that have the most significant impact on the number of infected cells and free virus particles, we conducted a sensitivity analysis. This analysis allowed us to determine the sensitivity of the basic reproduction number $R_0$ with respect to a specific parameter $\omega$ by calculating the derivative $\frac{\partial R_0}{\partial \omega }$. By plotting the dynamics of infected cells *I*(*t*) and free virus particles *V*(*t*) for various values of the infection rate $\beta$, we observed an interesting trend: when the infection rate was low, the number of infected cells and free virus particles exhibited a slower rate of decrease. Additionally, we found that the sensitivity of parameter $d_2$ and the latent period $\frac{1}{k}$ have a similar impact to $\beta$, which is demonstrated in Fig. 6.

**Figure 7:**
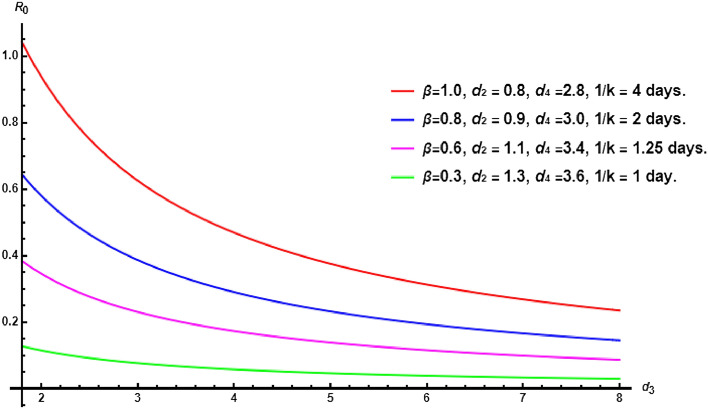
The basic reproductive number $R_0$ with respect to $d_3$ for different values of other parameters. Identifying $d_3$ as the most sensitive parameter highlights its significant influence on the within-host dynamics. This knowledge aids in prioritizing interventions targeting infected cells, potentially leading to improved patient outcomes by modulating $d_3$.

The death rate of free virus particles $d_4$ and the rate of free virus production *p* exhibit similar effects on the number of infected cells *I*(*t*) and free virus particles *V*(*t*), particularly impacting the abundance of free virus particles. However, the parameter that demonstrates the highest sensitivity to both *I*(*t*) and *V*(*t*) is the death rate of infected cells $d_3$. As we mentioned previously, this death rate represents a combination of natural clearance mechanisms and the role of the immune system. Therefore, it becomes essential to consider treatments that can reduce the infection within the host. Notably, increasing the death rate of infected cells leads to a decrease in the number of free virus particles, as depicted in Fig. 6. To further examine the influence of $d_3$, the most sensitive parameter, we plot the variation of $R_0$ with different values of other parameters in Fig. 7. This analysis provides valuable insights into the interplay between key parameters and their impact on the spread of the infection within the host.

Another control measure is the elasticity index which measures the change of $R_0$ with respect to the change in the parameters. The elasticity of $R_0$ with respect to the parameter $\omega$ can be calculated by $\Upsilon _{R_0}^\omega = \dfrac{\partial R_0}{\partial \omega } \times \dfrac{\omega }{R_0}$. Consequently,

$$\begin{aligned} \Upsilon _{R_0}^\beta = \Upsilon _{R_0}^p = 1, \, \, \, \, \Upsilon _{R_0}^{d_3} = \Upsilon _{R_0}^{d_4} = -1, \, \, \, \, \Upsilon _{R_0}^{k} = \frac{d_2}{d_2 + k}, \, \, \, \, \Upsilon _{R_0}^{d_2} = \frac{-d_2}{d_2 + k}. \end{aligned}$$

**Figure 8:**
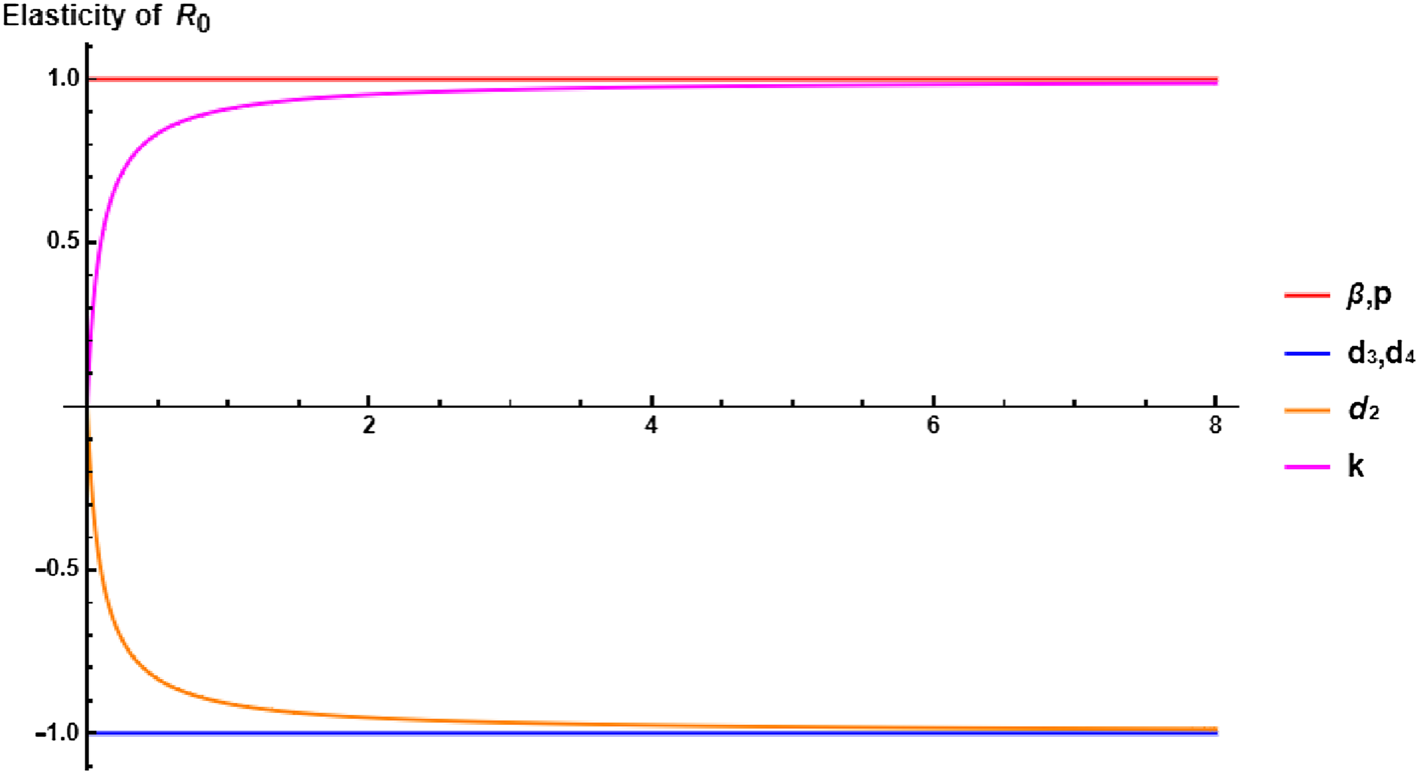
Elasticity of $R_0$ allows to assess the sensitivity of the basic reproduction number to variations in specific parameters.

The elasticity of $R_0$ exhibits positive correlations with respect to the parameters $\beta , p$ and *k*. Decreasing these parameters leads to a reduction in the value of $R_0$, indicating that the infection can be effectively eliminated from the human body. Conversely, negative correlations are observed with parameters $d_2$ and $d_3$. This implies that increasing the death rates of latent cells and infected cells, possibly through treatments such as plasma therapy, monoclonal antibodies, and immune system-boosting foods, can contribute to the eradication of the disease. These correlation relationships, highlighting the impact of parameter changes on $R_0$, are visualized in Fig. 8. Understanding these relationships is crucial for designing effective interventions and treatment strategies to combat the infection.

There is a positive correlation between $R_0$ and *T*(0) which means a larger initial number of target cells *T*(0) in a limited cell population can lead to a higher probability of infection and potentially faster virus spread.

Based on this discussion, there are additional real applications to the considered problem. Firstly, in Treatment Optimization, the sensitivity analysis identifying the death rate of infected cells as a critical parameter can inform the development of enhanced treatment strategies, leading to improved patient outcomes. Secondly, in Vaccine Development, the incorporation of latent class analysis into our model enables its use in assessing and refining COVID-19 vaccine candidates, aiding in the development and evaluation of effective vaccination strategies. Lastly, in Risk Assessment and Management, our model’s sensitivity analysis offers valuable insights into the relative significance of various parameters in shaping disease outcomes. This information can assist in conducting risk assessments, informing mitigation strategies, and efficiently allocating healthcare resources.

## Conclusion and further directions

In this study, we present a novel formulation of a within-host COVID-19 mathematical model. Unlike previous studies, our model takes into account the latent class, providing a new perspective on disease dynamics. We examine the nonnegativity and ultimate boundedness of the analytical solution to gain insights into the behavior of the system. Furthermore, we investigate the extinction and persistence of the disease within the human body by analyzing the local and global stability of equilibrium states, specifically $E_0$ and $E^*$. To support our findings, we perform numerical simulations and validate the results through the examination of sensitivity and elasticity of $R_0$ concerning the model’s parameters. Our results reveal that the disease dies out when $R_0 < 1$ without treatment, whereas it persists when $R_0 > 1$. Of notable importance is the death rate of infected cells, which emerges as a highly sensitive parameter that can be enhanced through appropriate medical therapy and a diet that supports the immune system. Additionally, we propose that our research can be expanded to incorporate discrete and distributed delays, further enhancing our understanding of the disease dynamics. Moreover, fractional models offer a more accurate representation of complex and heterogeneous systems. They capture the intricate nature of disease transmission, taking into account factors such as varying susceptibility, heterogeneous mixing patterns, and non-integer order derivatives^42,43^.

## Data Availability

All data used in this manuscript have been presented within the article.

## Code availability

The codes used and/or analysed during the current study available from the corresponding author on reasonable request.
